# Exploring the longitudinal dynamics of herd BVD antibody test results using model-based clustering

**DOI:** 10.1038/s41598-019-47339-6

**Published:** 2019-08-06

**Authors:** J. I. Eze, G. T. Innocent, K. Adam, S. Huntley, G. J. Gunn

**Affiliations:** 1 Department of Veterinary and Animal Science, Northern Faculty, Scotland’s Rural College (SRUC), An Lòchran, 10 Inverness Campus, Inverness, IV2 5NA United Kingdom; 2 Biomathematics and Statistics Scotland (BioSS), JCMB, Edinburgh, EH9 3FD United Kingdom

**Keywords:** Viral infection, Risk factors

## Abstract

Determining the Bovine Viral Diarrhoea (BVD) infection status of cattle herds is a challenge for control and eradication schemes. Given the changing dynamics of BVD  virus (BVDV) antibody responses in cattle, classifying herds based on longitudinal changes in the results of BVDV antibody tests could offer a novel, complementary approach to categorising herds that is less likely than the present system to result in a herd’s status changing from year to year, as it is more likely to capture the true exposure dynamics of the farms. This paper describes the dynamics of BVDV antibody test values (measured as percentage positivity (PP)) obtained from 15,500 bovines between 2007 and 2010 from thirty nine cattle herds located in Scotland and Northern England. It explores approaches of classifying herds based on trend, magnitude and shape of their antibody PP trajectories and investigates the epidemiological similarities between farms within the same cluster. Gaussian mixture models were used for the magnitude and shape clustering. Epidemiologically meaningful clusters were obtained. Farm cluster membership depends on clustering approach used. Moderate concordance was found between the shape and magnitude clusters. These methods hold potential for application to enhance control efforts for BVD and other infectious livestock diseases.

## Introduction

Bovine viral diarrhoea (BVD) is an economically important disease of cattle caused by a pestivirus^1^. National approaches to BVD control vary widely^2^ and while the virus is endemic throughout most of the world, a small number of countries have successfully eradicated BVD or implemented national control schemes^3^. BVD virus (BVDV) is present in the United Kingdom, but Scotland has implemented an eradication programme since 2010^4^.

Antibody enzyme linked immunosorbent assay (ELISA) BVD diagnostic tests on blood or milk samples are used commonly to provide data about BVD exposure, both in individual animals and at a herd level. Previous studies have examined genetic clustering of BVD virus^5^, BVD spatial clusters^6,7^ and seroprevalence cohorts among young stock in Scottish suckler herds^8^. These studies indicate that distinct subgroups of BVD infected farms can be identified although little is known about how these subgroups change over time. Herd BVD status can also be determined by antibody testing of bulk milk tank samples on dairy farms^9^, demonstrating the utility of antibody testing of samples from adult cows in classifying herds’ BVD status. In this study, blood samples were tested for BVDV antibody using a commercially available indirect ELISA test (SVANOVA) kit in accordance with the manufacturer’s instructions^10^. The test results were presented as percentage positivity (PP) values, calculated from the optical density (OD) values of the samples in comparison to positive and negative control samples^8,9^. We used these PP values as proxy measures of BVDV antibody levels in this paper. We believe that there is a monotonic relationship between PP values and antibody levels but this may not be linear. Also, our interest is in the patterns of change over time rather than the numerical value at any one time point.

Given that BVDV antibodies are dynamic due to their response to exposure factors, it is likely that cluster memberships (or status groups) obtained from a single cross-sectional antibody test may change if clustering is repeated after another test in the near future. However, clustering BVDV antibody longitudinal trends (trajectories) may give a truer picture of cluster membership as they are more likely to capture the infection dynamics of each cluster member over time. Therefore, patterns of farm antibody trajectories or patterns of changes of farm BVD status over time may be important in monitoring impact of control measures adopted to eradicate BVD. Clustering these patterns may reveal a group of problem farms where targeted control efforts and resources should be directed. This is likely to be more robust than doing so based on data from single time points. Therefore, the goal of this paper is to propose a model-based methodology that clusters farms based on the pattern of longitudinal trends in farm-level test results.

Model-based methods for clustering longitudinal data have been applied successfully to the clustering of gene expression time course data^11–17^. Genolini *et al*.^18^ extended the k-means clustering to the context of shape-respecting partitioning. It has been shown that model-based longitudinal clustering methods out-performed other clustering methods^19,20^. The beauty of the approaches used in our paper is their ability to model dependence of the model mixture parameters on time and this dependence may be linear or nonlinear^13,21,22^.

This paper aims to: describe the average levels of BVDV antibody, measured as PP values, and their changes over time on each farm; apply simple linear models on each farm’s antibody trajectory to compare the rate of change between farm groups; use model-based longitudinal clustering methods to group farms that displayed similar magnitudes and similar shapes of PP trajectories, respectively. It also studies the epidemiological commonalities between farms in the same cluster and explores the agreement between the magnitude and shape clustering.

We note that there is no unique approach of clustering any given dataset as there may be more than one quantitative characteristic of the data on which clustering could be based. Different approaches will likely yield different clusters. The use of these three approaches in this paper is aimed to cover a spectrum of possible epidemiological questions where interest may be either on the levels, trends or shapes of an epidemiological phenomenon over time. We shall discuss the pros and cons of each of these approaches and assess the extent of agreement between cluster partitions obtained using the model-based shape and magnitude clustering.

## Results

### Analysis of magnitude

**Figure 1:**
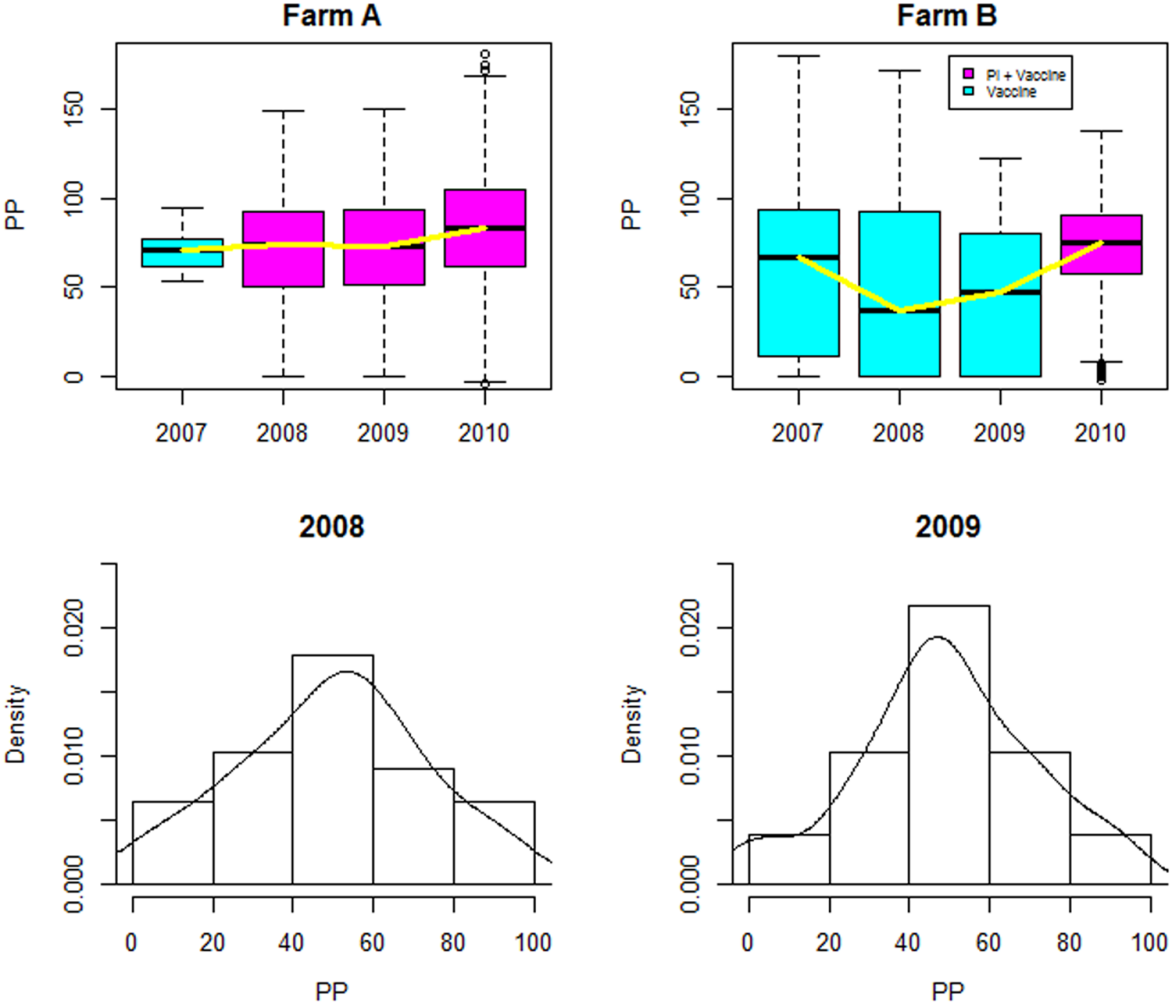
Distribution of within farm BVDV antibody PP values in two farms (first row). The second row shows the distribution of mean farm antibody PP values for all farms in 2008 and 2009 respectively. The plots indicate that antibody PP values can be approximated by a normal distribution.

The results of BVDV antibody tests are often interpreted as positive or negative on the basis of established cut-off values for the PP. This study, however, used the quantitative PP values to provide more nuanced information about the magnitude of antibody test value across all cattle in the herds sampled. Analyses indicate that patterns of antibody response to BVD virus and/or vaccine exposure varied between farms. We show two examples in the first row of Fig. 1 which represents the outcome of preliminary analysis for two farms. For confidentiality, the farms are referred as A and B. Farm A is a large dairy farm with an average of 630 cattle tested for BVD each year between 2007 and 2010. Vaccination was implemented from 2007 through 2010 and PI animals were detected and removed from the farm in 2008, 2009 and 2010. The exposure to BVD virus and vaccination may explain the increasing variation in BVDV antibody PP values with increasing year in the farm. Farm B is a beef farm where an average number of 206 cattle were tested per year over the four year period. A vaccination program was carried out on this farm from 2007 and in 2010 a PI animal was detected and removed. The graph of Farm B in Fig. 1 appears to suggest (given the increase in 2009) that the PI may have been introduced onto the farm at some point in 2009 but detected in 2010. Light blue boxplots in Fig. 1 indicate that the herd was vaccinated while magenta indicate that the herd was vaccinated and PI bovine(s) detected in the given year. Average PP levels were slightly higher in farm A compared to farm B. The differences in exposure to PI animals could be responsible for the difference in antibody levels in the two farms. The yellow line depicts the change in the median antibody PP levels over the study period.

The graphs in the second row of Fig. 1 represent the distribution of average BVDV antibody PP values for all thirty-nine farms in 2008 and 2009 respectively. These graphs suggest that the data can be approximated by a normal distribution.

### Trend clustering

**Figure 2:**
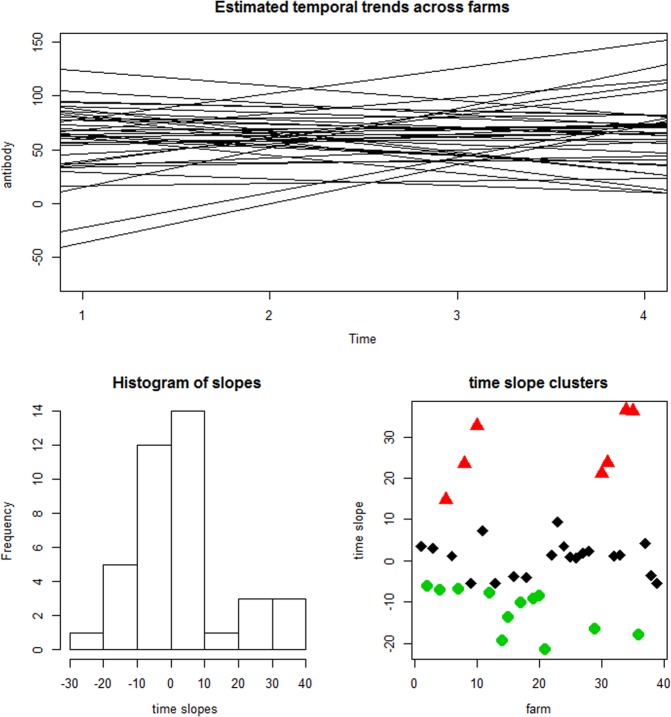
First row: Average linear time trend of the farm-level BVDV antibody PP trajectories. Second row: The histogram of temporal slopes and three distinct clusters of the temporal slopes indicated by the different colours for group of farms within each cluster.

The first stage at examining the shape of the trajectories was to assess the overall linear trend of changes in mean PP values over time on each farm. The graphs in Fig. 2 show the outcome of fitting the linear trend model (Eq. 1) to the PP value trajectory for each farm. The graph on the top row is the plot of the model estimates of temporal trend for each farm. Three trending groups are identifiable from this plot: farms with stable PP values, farms with decreasing and farms with increasing PP levels. The distribution of the slopes (rate of change) of mean PP values over time across farms is represented by the histogram.

Although the slopes are either negative (18 farms) or positive (21 farms), the three trending groups, captured by the steepness of the slopes, are discernible from the histogram. Implementing formal statistical clustering of the slopes confirms the existence of the three clusters as shown in second row of Fig. 2.

The rates of change vary within the increasing (range of slope: 1 to 40) and decreasing (range of slope: −30 to −1) groups of farm. Therefore, the rate of improvement or deterioration was different for different farms.

### Clustering by magnitude

**Figure 3:**
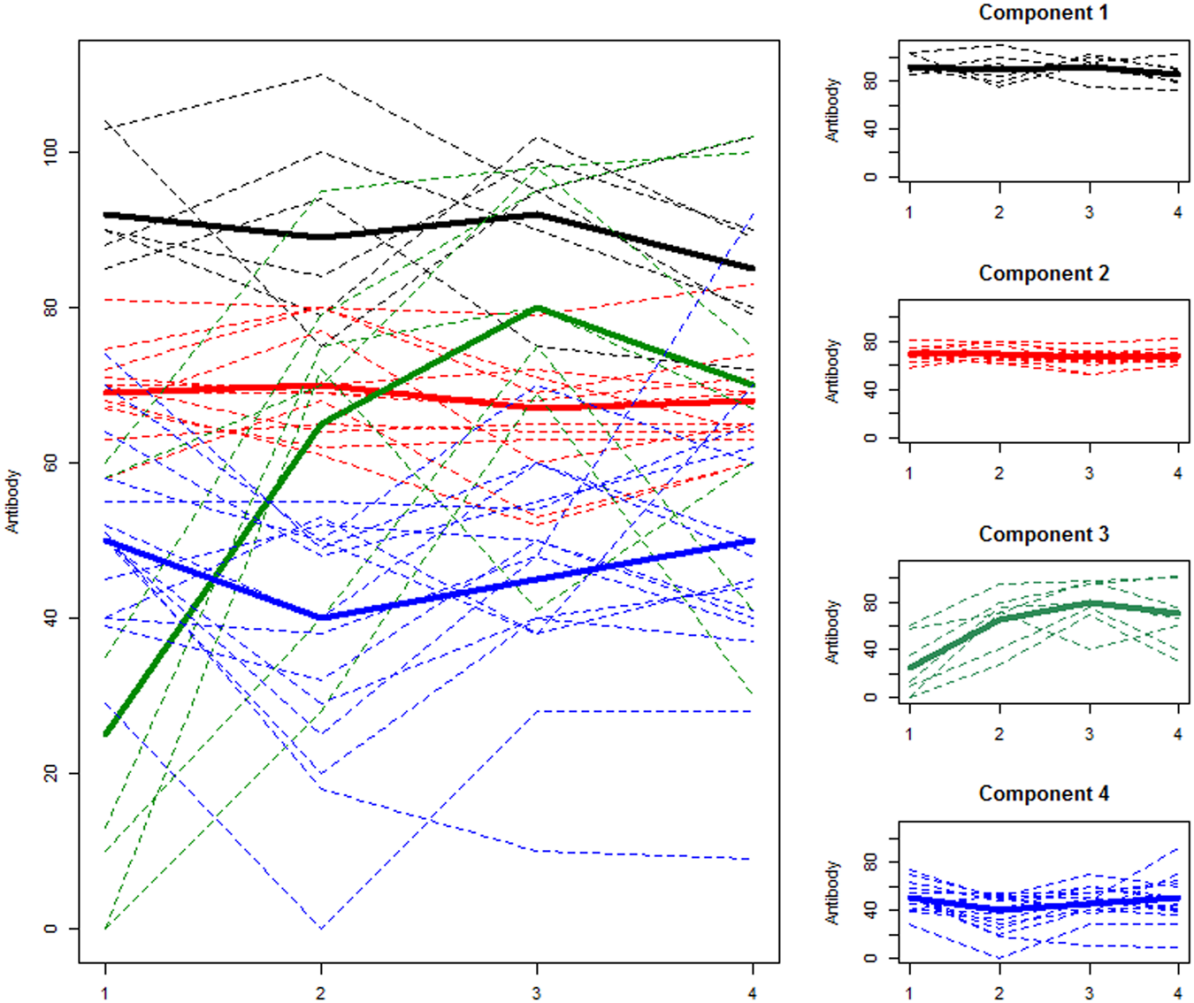
Longitudinal clusters of mean farm level BVDV antibody PP values classified by their magnitude. The dotted lines are individual farm trajectories and bold lines are mean of the trajectories in each component. The x-axis represents the four time period of the study (2007–2010) while the y-axis is the farm mean PP value.

Table 1 and the plots in Fig. 3 depict the cluster of the farms by the magnitude of their longitudinal mean PP values with little distinction between the temporal shapes of the trajectories.

**Table 1:** Summary of clustering by magnitude of farm PP trajectories.

Component	Mean PP	Range of trends of mean PP	No. of farms (%)
1. High levels	89.3	85–92	6 (15.3)
2. Medium levels	68.0	67–70	12 (30.8)
3. Increasing levels	59.8	25–79	7 (18.0)
4. Relatively low levels	45.9	37–51	14 (35.9)

Four clusters were identified in our data using this approach - one group of farms where antibody levels remained high through the study period (component 1); one group of farms with medium levels throughout (component 2), one group where antibody levels broadly increased over the study period (component 3) and one group that maintained comparatively low antibody levels (component 4). Table 1 shows the range of mean values within each cluster. The hierarchy in the magnitude of antibody levels among the components is very evident in the table. The higher the magnitude of BVDV antibody PP values, the greater the likelihood of prior exposure to BVD infection and/or vaccination. Therefore, risk levels are higher among farms in the top two components. Also, component 3 represents a group of farms where the BVD infection situation appeared to worsen over time.

### Shape analysis

The approach used to obtain Fig. 3 appears to be driven by magnitude such that trajectories with similar shapes but with differing antibody magnitudes were grouped in different clusters. In order to understand patterns of change over time and groups with similar change pattern, a consideration of the shapes of the trajectories was employed in the clustering process.

### Shape clustering

**Figure 4:**
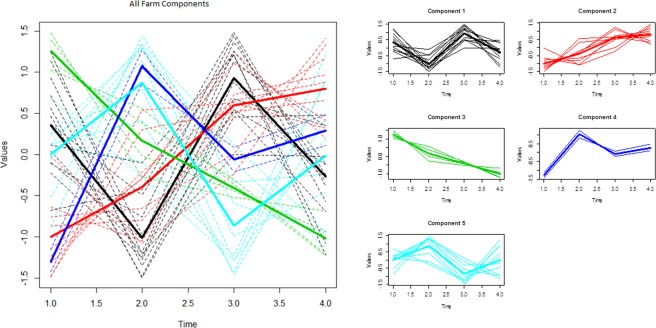
Longitudinal clusters of the shapes of farm antibody PP trajectories with cluster means fitted as nonlinear function of time. The dotted lines are individual farm trajectories; bold lines are mean of the trajectories in each component. The x-axis represents the four time period of the study (2007–2010), y-axis are the standardized (shape) values of the antibody PP values.

The two clustering approaches considered in the preceding sections conceal some useful patterns which may be of epidemiologic interest. Figure 4 shows the clustering of farm trajectories based only on the shape of their curves. The component means were modelled as a nonlinear function of time in order to capture the shapes of the trajectories. Five distinct shape clusters were identified: Component 3 is a group of farms where antibody PP values declined consistently over the study period while component 2 is a group with increasing mean PP values over time. On the other hand, components 1, 4, and 5 represent changing PP values at different time points which may reflect responses to exposure and/or control measures. This approach is useful where interest is not only to establish the shape or temporal pattern of the trajectories but also to determine or visualise the point in time when change occurred. Extraction of the shape of the trajectories makes the point of change very distinct.

### Correspondence between magnitude and shape clusters

Table 2 shows the correspondence between the clusters obtained from the magnitude and shape clustering. It can be seen from the table that there is a match between cluster 2 of the shape and cluster 3 of the magnitude clustering. This is because farms in the two clusters have increasing trend over the four year study period. The estimate of adjusted Rand index based on Table 2 gave a correspondence of 4 in 10. This means that the two clustering methods agree in four out of ten times that a random pair of farms should either be in the same cluster, or in different clusters. The two clustering methods appear to agree mostly where farm trajectories are linearly shaped. This is an interesting result as it confirms that the two clustering methods represent different BVDV antibody dynamics.

**Table 2:** Correspondence between antibody magnitude and shape cluster memberships.

Shape clusters	Magnitude clusters				No. of Farms
	Cluster 1	Cluster 2	Cluster 3	Cluster 4	
Cluster 1	3	4		6	13
Cluster 2			6	3	9
Cluster 3		1		3	4
Cluster 4		2	1		3
Cluster 5	3	5		2	10
No. of Farms	6	12	7	14	39

### Insight into the clusters

Examination of membership of the magnitude clusters (shown in Table 1) reveals that all the six farms in component 1 (the farms with the highest mean antibody PP values) vaccinated, and three of them also had at least one PI animal within the study period. Eight of the twelve farms in component 2 (medium mean antibody PP values) had held PIs. Six out of the seven farms in component 3 (with rising antibody PP values) had been vaccinated and/or contained a PI. Component 4 is made up of a variety of farms. The presence of a PI animal allows us to infer that, at that point, there is circulating BVD virus on the farm. However, it does not allow us to make any inference about when the virus entered the farm or when/if it stopped circulating. We therefore do not try to associate the specific timing of identification of a PI animal with any characteristic of the antibody profile, but merely infer the fact that it occurred during the sampling timeframe might provide information which helps to profile clusters. Antibody levels may be associated with production type. All six farms in component 1 are beef farms and of the eleven dairy farms, six belonged to the relatively low antibody group (component 4). We can subjectively deduce that the epidemiological commonalities between farms in each component are: vaccinated group (component 1), PI group (component 2), vaccine + PI group (component 3) and component 4 is mixed.

The majority (62%) of farms in component 1 of the shape clusters (Fig. 4) were farms which had vaccinated but had no history of having held PI animals and a further 23% had no known influence of vaccination and PI presence. This suggests natural and vaccine effect fluctuations in BVDV antibody PP values. Management information is available for eight out of the nine farms in component 2. Of the eight, Six (75%) of the farms in component 2 with increasing antibody PP values were farms that had the simultaneous effects of the presence of PI and vaccination. The two remaining farms only vaccinated. Of the farms with decreasing antibodies in component 3, 75% were farms with no history of having held PI animals or vaccination in the study period. 80% of the farms in component 5, had PI animals mostly in the first and second year (2007/2008) of the study. Therefore, the epidemiological commonalities that describe the clusters can be deduced as: vaccine group (component 1), vaccine + PI group (components 2 and 4), no exposure group (component 3) and PI group (component 5).

Hence, farms with high or increasing BVDV antibodies were either vaccinated and/or had PI animals. This suggests that vaccination can lead to increases in BVDV antibody levels but simultaneous exposure of farms to the effects of both vaccine and BVD virus is likely to have a more marked effect on the levels of BVDV antibody.

## Discussion

The dynamic nature of BVDV antibody levels means that herd BVD status assigned on the basis of the results obtained from a single cross-sectional antibody test in youngstock is likely to change if the herd is tested over time. Therefore, the dynamics in the levels of BVDV antibodies, when measured over time, can mean that the BVD status (whether BVD is present on farm or not) is likely to change as antibody levels change if herd status is based on BVDV antibodies alone. We posit that classifying farms based on the longitudinal behaviour of their BVDV antibody levels could offer a novel, complementary approach for grouping farms as it is more likely to capture or represent the true infection experience or exposure dynamics of the farms. This may also reflect the dynamics of management changes because the differences in exposure and control measures adopted by the farms may result in antibody longitudinal patterns and magnitudes differing between farms. Using antibody PP value as an proxy measure of antibody level, our interest was to use advanced statistical methods to group farms with similar antibody magnitude, trend and shape, respectively. Each of these has implications for BVD control - magnitude gives an indication of exposure intensity, trend indicates general direction of change and shape indicates not only the direction of change, but also other complex nonlinear patterns which may be influenced by frequency of directional changes in time.

The results of the analyses indicate that BVDV antibody PP values vary substantially, both between and within herds as a result of BVDV exposure and/or vaccination. The analyses demonstrate the existence of distinct subgroups of farms in terms of BVDV antibody response patterns, as demonstrated by the clusters identified in our data. The resulting subgroups were a function of the methods and data characteristics on which clustering was based. The groups of farms isolated by these clustering methods are likely to require different BVD control interventions.

Analysis of the slopes revealed three trending groups in our data. Farms with near-flat slopes may represent farms with fairly stable mean BVDV antibody PP levels over time. Negative slope depict farms with decreasing levels of mean antibody PP levels indicating either exposure to BVD virus has stopped over time or cessation of a vaccination policy. Farms with positive slopes represent farms with increasing mean antibody PP levels indicating deterioration or worsening infection situation and/or sustained vaccination over time. The rate of improvement or deterioration was different for different farms. This method can be used as a naïve classification of farms where interest is in identifying the rates of response of farm groups to control measures or treatment. However, the method has the limitation of forcing linearity on all trajectories.

Clustering by BVDV antibody magnitude partitioned farms based on their longitudinal mean antibody levels. The magnitude of BVDV antibody levels, measured by PP values, gives an indication of the exposure intensity of the farm to BVD virus and/or vaccination. Hence, clustering farms by the magnitude of their mean PP values, groups together farms with similar levels of exposure to virus and/or vaccine. This method is useful when interest is to isolate high risk farms for closer scrutiny or for more in-depth investigation of BVD infection dynamics in herds with problematic, on-going infections. The ability of this method to partition farms by the magnitude of their longitudinal mean antibody PP values suggests that it could be employed in the assessment of any control measure aimed at reducing BVD infection (where antibodies are monitored over time). However, clustering by antibody magnitude masks some salient information that may be of interest as the clustering is dominated by the magnitude of PP values, such that farms with similar patterns but different antibody magnitudes were grouped in separate clusters. High variability in the antibody levels can lead to large dissimilarity among antibody trajectories with similar shape resulting in classifying similarly shaped trajectories in different clusters^23^. Also, extra information contained in the trajectories is swamped by the overall mean in each component (shown as bold lines). Consequently, the means within each cluster in Fig. 3 do not represent any individual farm trajectory since they are the means of differently shaped trajectories with similar magnitude. Therefore, it is not possible to assess changes in a given farm’s antibody PP values over time from the cluster means. For instance, a variety of behavioural patterns (stable, increase or reduction in antibody PP levels) over time is discernible within each component of Fig. 3.

Where interest is in the pattern of responses with no emphasis on the magnitude of antibody levels such that we would like to group farms with similar response patterns, we advocate the use of shape clustering as it can show explicitly the change patterns, points at which changes occur and groups of farms with similar patterns. However, the use of this approach entails the elimination of the magnitude. But the ability of the methods to group farms by their trending groups makes them a useful tool for the assessment of success or otherwise, of any management, intervention or control measures monitored over time.

The methods described in this paper do not provide a definitive herd status, but may help to identify high risk herds for further investigation. These approaches have the potential to enable the identification of groups of farms where control efforts appear effective and those to which more efforts should be directed. Shapes of temporal changes may be driven by factors that are of importance to BVD control programs. The identification of the direction, over time, of effect of these factors on BVDV antibody level may be very useful to assess the extent of success or otherwise of control programs. Subject to data availability, longitudinal patterns of antibody levels could offer an alternative or complementary approach to the present methods for classifying herds based on their BVDV antibody prevalence. It is appreciated that repeated measures of individual animal antibody levels could often seem an expensive option (although the overall trend is to cheaper tests) but for problematic herds, say towards the endgame of a regional control programme, it may actually be of significant benefit. This may be useful in herds where a PI animal is not in the herd, but infection is present, possibly from contact with an external source of infection, PI births may be rare but non-PI infection in the herd still relatively common. This might be the case in refractory farms, and an antibody approach might be more informative as it is based on more common occurrences.

Although we believe that the methods presented are likely to be useful in the eradication of BVD virus, particularly in refractory herds, our analysis is based on a small sample of farms. If the approach were to be widely adopted more information would become available such that the procedure could be validated. Furthermore the effect of sampling a subset from each farm could be investigated which would reduce costs to the farmer.

### Application

Longitudinal whole-herd testing for BVDV antibody may provide a more complete picture of BVD exposure within a herd than routine spot testing of a sample of youngstock as currently required by Cattle Health Certification Standards (the regulatory body for herd health schemes in the UK and Ireland) to demonstrate freedom from BVD although it substantially increases the costs associated with testing. However, repeated, whole-herd blood sampling for antibody testing is conducted routinely in beef suckler cattle as part of control programmes for diseases including paratuberculosis (Johne’s disease), and therefore represents a feasible strategy to obtain additional data to classify herds on the basis of their BVD status. If preliminary spot testing does not deliver a conclusive herd status, it may be used as part of a more in-depth assessment of BVD exposure within the herd. As this was an observational study using data obtained from commercial farms, it is possible that BVD exposures occurred in the study herds which were not captured in the epidemiological data.

The modelling methods described are a novel approach to exploring longitudinal patterns of antibody test results in animal populations, and shows potential for application to large datasets of BVD test results, such as herd health schemes, eradication schemes or national screening programmes, to monitor progress over time. The method also shows some potential for distinguishing vaccinated from infected herds, which is a major challenge in interpreting antibody test results for BVD.

The method described is generic and could be applied in a variety of scenarios where routine measurements (continuous, ordinal or binary outcomes) are collected over time and where interest is to distinguish groups with similar response pattern – for instance antibodies to other diseases, animal growth, milk quality, etc. - in order to understand the grouping factors. Therefore, all that is required to apply these methods is that data be collected longitudinally with the objective of investigating change patterns with the view of grouping entities with similar longitudinal patterns.

## Conclusions

This paper describes a novel application of a modelling method using the example of determining the dynamics of Bovine Viral Diarrhoea (BVD) infection status of cattle herds as a challenge related to disease control and eradication programmes. At present, determination of a herd’s BVD status is based on the outcome of antibody tests conducted on samples from a small number of young animals to assess recent exposure. However, dynamic changes in the levels of BVDV antibody in adult cows, measured over time, can mean that BVD status is likely to change as antibody levels change. This study shows that distinct longitudinal clusters of farm BVDV antibody PP values exist and that model-based longitudinal clustering could help to capture farms with similar  history of infection or exposure dynamics over time. Different cluster memberships were obtained depending on the type of clustering approach used. However, moderate concordance was found between the shape and magnitude clusters suggesting that they represent different antibody dynamics. Longitudinal herd tests could offer a complementary approach to the current method of youngstock antibody spot tests and tests for virus in defining herd status for the purposes of control and eradication programmes. As anticipated, higher antibody levels and rising antibody levels are frequently associated with the presence of a PI animal or with vaccination but this expected response is far from universal. Further work is required to explore the factors associated with variation in serum antibody levels in adult cattle.

## Methods

Data were collected between 2007 and 2010 from thirty-nine cattle farms located in Scotland and Northern England, comprising eleven dairy and twenty-eight beef farms. Study herds were established in association with nine veterinary practices who identified herds within their clientele that either had ongoing or had recently encountered problems with BVD virus infection^24^. Therefore, recruited farmers were those who perceived BVD as a problem and were looking for assistance in controlling BVD. All sampling of cattle was conducted by veterinary surgeons as part of ongoing BVD eradication/control programmes on the participating farms, in accordance with the Veterinary Surgeons Act 1966. As the samples were collected for clinical rather than experimental purposes, licensing under the Animals (Scientific Procedures) Act 1986 was not required. The manuscript presents further retrospective analysis of the results obtained from these clinical disease investigations. Serum samples were collected annually from all animals on the study farms. A total of 27,790 samples from 15,500 bovines were collected throughout the study period. However, only test results from over 9800 adult cows (over 12 months of age to exclude maternal antibody) were used for clustering analyses. The study was conducted prior to the introduction of Scottish BVD eradication scheme and the interventions for BVD control on study farms focused on identification and culling of persistently infected (PI) animals and the use of vaccination as a control strategy on some farms. Farm management information was collected using a questionnaire and recorded a face-to-face interview with the farm vet.

Samples were tested for BVDV antibody using a commercially available indirect ELISA test kit in accordance with the manufacturer’s instructions^10^. The test results were presented as percent positivity (PP) values, calculated from the optical density (OD) values of the samples in comparison to positive and negative control samples as:

$${\rm{P}}{\rm{P}}=\frac{100\ast ({\rm{O}}{\rm{D}}\,{\rm{s}}{\rm{a}}{\rm{m}}{\rm{p}}{\rm{l}}{\rm{e}}-{\rm{O}}{\rm{D}}\,{\rm{n}}{\rm{e}}{\rm{g}}{\rm{a}}{\rm{t}}{\rm{i}}{\rm{v}}{\rm{e}}\,{\rm{c}}{\rm{o}}{\rm{n}}{\rm{t}}{\rm{r}}{\rm{o}}{\rm{l}})}{{\rm{O}}{\rm{D}}\,{\rm{p}}{\rm{o}}{\rm{s}}{\rm{i}}{\rm{t}}{\rm{i}}{\rm{v}}{\rm{e}}\,{\rm{c}}{\rm{o}}{\rm{n}}{\rm{t}}{\rm{r}}{\rm{o}}{\rm{l}}}$$

The results of BVDV antibody tests are often interpreted as positive or negative on the basis of established cut-off values for the PP^8,9^. This study, however, used the quantitative PP values to provide more nuanced information about the longitudinal patterns of antibody response in the cattle sampled. Mean PP values of all adult cows tested on a given farm in a particular year were used for the clustering analyses. Preliminary analysis suggests that the distribution of the PP values on each farm and each year can be approximated by a normal distribution (Fig. 1). Some farms joined the study a year later; consequently, they had no antibody information in 2007. For such farms where no animal was sampled in a given year within the study period, imputation of the farm mean antibody PP value for that year was done by generating random values from the normal distribution using the overall mean and standard deviation of that farm’s BVDV antibody PP values across all the years as parameters of the distribution.

Clustering of the longitudinal values of PP were performed in three ways based on linear trends, levels (or magnitude) and shapes of their curve as outlined below:

### Clustering by linear trend

By default, most longitudinal data are analysed by imposing linearity assumptions on the outcome data. In order to understand the general trend pattern in each farm, a simple linear model (Eq. 1), was fitted to each farm’s mean antibody PP trajectory. The slopes obtained from the models were clustered using k-means approach^25^ to check for similarities in trending patterns among the farms. This method serves as a form of transformation that brings all the trajectories to the same form and provides preliminary information on possible trending groups. It is a naïve examination of groupings of farms by their linear trends for classification purposes and not intended for prediction of antibody levels. The simple linear model fitted to extract the trend from each farm’s data is given as:

1 $${y}_{it}={\alpha }_{i}+{\beta }_{i}t+{\varepsilon }_{it}$$

where *y*_*it*_ is the mean antibody PP value in farm *i* at *time t*. α_*i*_, *β*_*i*_ are the intercept and slope for farm *i* respectively and ${\varepsilon }_{it}\,\sim N(0,{\sigma }_{i}^{2})$ is the random error term. The plot of estimates of average trend given by $\hat{y}=\hat{\alpha }+\hat{\beta }t$ for each farm gives a graphical representation of aggregate trend patterns across farms.

This analysis gave the impetus to further seek out ways of capturing the nonlinear pattern of some farms’ longitudinal trends: the shape cluster analysis.

### Clustering by antibody magnitude and shape

Trends in levels and shapes may provide distinct information that is likely to elicit different management actions. We define antibody levels or magnitude as the actual PP value. Antibody magnitude (level) gives an indication of exposure intensity. Shape indicates the direction of change and other complex nonlinear patterns influenced by the frequency of directional changes over time. Therefore, we consider the partitioning of a measure of farm BVDV antibody trajectory by similarity in trends in their levels and shapes. To achieve this, we use models that not only recognise patterns but also can group similar patterns together. We used model-based clustering approach which considers data as coming from a probability distribution that is a mixture of two or more clusters. Each cluster has a density function and an associated weight in the mixture (or probability of being part of the mixture). The approach does not use distance measures for allocating data to clusters but computes the probability of each trajectory belonging to each cluster. Given that our data is longitudinal and we are interested in clustering a longitudinal measure of BVD antibody for each farm, it is important to model the serial correlation between measurements at different time points within each farm. This is done by defining an appropriate covariance structure to account for this correlation. This procedure is explained in detail below.

### Clustering by magnitude

The second part of analysis focused on clustering farms based on the magnitude of their mean antibody profiles over the time period. This clustering enabled the distinction of different groups based on antibody magnitude in order to identify farms that started with high antibody levels at baseline and remained high through the study period, those with median average values over time and those that started with low levels and remained low. It was also of interest to distinguish farms whose antibody levels increased or decreased on average over time.

For magnitude clustering, data were modelled as a mixture of multivariate Gaussian-distribution with density given as:

2 $$f(y|\theta )=\,\sum _{k=1}^{K}{\pi }_{k}\,{f}_{k}(y|{\theta }_{k})$$

where y are the trajectories of farm mean antibody PP, *f*_*k*_ and *θ*_*k*_ are the density and parameters of the *k*th component or cluster and *π*_*k*_ is the mixing proportion with the condition that $\,{\pi }_{k} > 0,\,\,such\,that\,\sum _{k}^{K}\,\pi =1$.

3 $${\hat{z}}_{ik}=\frac{{\hat{\pi }}_{k}\,f(y|{\theta }_{k})}{{\sum }_{k=1}^{K}\,{\hat{\pi }}_{k}\,f(y|{\theta }_{k})}$$

Estimate of *a posteriori* probability, ${\hat{z}}_{ik}$, is the probability that *i*th farm trajectory belong to cluster *k*. Hence, each observed trajectory belongs to every cluster with some probability. Trajectories were assigned to clusters where they have the maximum *a posteriori* probability. The analysis was conducted in R statistical software^26^ using Longclust which implements clustering using multivariate Gaussian mixture models with Cholesky decomposed covariance structure^27^. This enables the estimation of the correlation between measurements at different time points. The Expectation Maximisation (EM) algorithm was used to obtain maximum likelihood estimates of the model parameters and the Bayesian Information Criterion (BIC) was used for model selection. For further details of this approach interested readers should see^12,28^.

### Clustering by shape alone

The downside to clustering by magnitude is that magnitude of antibody PP tends to dominate the grouping such that little consideration is given to the shape of the trajectories especially where the shape is nonlinear. Consequently, farms with different shapes can be grouped together if they have similar average antibody magnitude over the time period.

The third part of the analyses investigated the clustering of trajectories with similar shapes after eliminating the dominant effect of the magnitude of antibodies. It focused explicitly on clustering shapes of the trajectories in order to compare groups of farms based on their antibody temporal change. In order to remove the effect of antibody magnitude and variability, so as to extract the shape of each trajectory, each farm’s longitudinal data was standardized by subtracting the farm’s overall mean antibody PP value and dividing by the overall standard deviation for that farm. Subtracting the mean eliminates the magnitude and dividing by the standard deviation removes the effect of variability. Thus:

4 $${y}_{it}^{\ast }=\frac{{y}_{it}-{\mu }_{i}}{{\sigma }_{i}\,}$$

and

5 $$f({y}^{\ast }|{\theta }^{\ast })=\sum _{k=1}^{K}\,{\pi }_{k}^{\ast }{f}_{k}({y}^{\ast }|{\theta }_{k}^{\ast })$$

${y}_{it}^{\ast } \sim \,N(0,1)$ defines the shape of each farm’s trajectory, *y*_*it*_ is farm *i* mean BVDV antibody PP value at time *t*, *μ*_*i*_ and σ_i_ are the overall mean and standard deviation of the farm i’s antibody PP values. The density and parameters of the clusters and the a posteriori probabilities are as defined in Eqs 2 and 3 above. This transformation imposes the constraint that variability and magnitude are the same for each trajectory and by extension, for each cluster. Hence, with this operation, the only difference between any two trajectories was the difference in their shapes. Therefore, clustering the standardized trajectories is expected to produce clusters with similar shapes.

### Comparison of magnitude and shape clustering methods

The extent of agreement between clusters obtained when farms were clustered by magnitude and by shape of the trajectories was assessed using Rand Index^29^ which estimates the proportion of the number of times a pair of farms is grouped together and the number of times a pair of farms is not grouped together by the two clustering methods. Hence, if the two clustering methods group any two farms together in a given cluster, this implies that the two agree that both farms should be together. Also if both methods agree that any pair of farms should be in different clusters, this also is counted as agreement between the two methods. The value of the index ranges from 0, indicating that the two clustering methods do not agree, to 1 indicating that the two clustering are exactly the same. However, the index is influenced by the number of clusters - it increases with number of clusters. Consequently, due to this limitation, we adopted its adjusted version proposed by Hubert and Arabie^30^ which adjusts for the expected number of random agreements and assumes a generalised hypergeometric distribution under the null hypothesis that the two clustering were drawn randomly.

## Data Availability

Data used in this study was collected under confidentiality agreement with each farm. Access to the data would need additional permission from the farms. Anyone interested in using the data should contact the corresponding author.
